# A Chemically Induced Experimental Colitis Model with a Simple Combination of Acetic Acid and Trinitrobenzene Sulphonic Acid

**DOI:** 10.5152/tjg.2022.22174

**Published:** 2023-03-01

**Authors:** Havva Yasemin Cinpolat, Güler Buğdaycı, Neriman Şengül, Hesna Müzeyyen Astarcı

**Affiliations:** 1 Department of Medical Biochemistry, Çanakkale Onsekiz Mart University Faculty of Medicine, Çanakkale, Turkey; 2 Department of Medical Biochemistry, Abant İzzet Baysal University Faculty of Medicine, Bolu, Turkey; 3 Department of General Surgery, Abant İzzet Baysal University Faculty of Medicine, Bolu, Turkey; 4 Department of Pathology, Lokman Hekim University Faculty of Medicine, Ankara, Turkey

**Keywords:** Acetic acid, inflammatory bowel disease, interleukin 33, ST2, trinitrobenzene sulphonic acid

## Abstract

**Background:**

It was aimed to induce a new experimental colitis model by using acetic acid and trinitrobenzene sulphonic acid together and to investigate the severity of inflammation biochemically and histopathologically in comparison with other models.

**Methods:**

Fifty-six Wistar albino male rats were randomly divided into 4 groups as control, acetic acid, trinitrobenzene sulphonic acid, and combined groups, and the animals were sacrificed following the induction of colitis on the third day and on the seventh day. The serum amyloid A and myeloperoxidase were tested in plasma samples, and the tumor necrosis factor-alpha, interleukin 33, and ST2 were assayed in colon tissue samples with enzyme-linked immunosorbent assay in addition to histopathological examination.

**Results:**

There were statistically significant differences between the combined and the control groups both on the third day and on the seventh day in all parameters. There was no difference between the acetic acid group on the seventh day and the control groups in biochemical parameters.

**Conclusions:**

The acetic acid model forms acute colitis. The combined model is found to be more successful in forming inflammation when compared to other models.

**Main Points**

- Acetic acid induces a local inflammation in the colon; however, this does not evolve into a systemic inflammation and the colitis is of acute nature.
- The trinitrobenzene sulphonic acid model is a successful model in inducing inflammation, but it mostly induces colitis of chronic effect.
- The combined model also resulted in systemic inflammation in addition to causing inflammation in the colon and had success in generating colitis both during acute and the chronic period.

## Introduction

Ulcerative colitis (UC) and Crohn’s disease (CD), referred to as inflammatory bowel disease (IBD), are recurrent intestinal pathologies manifested by chronic inflammation of the intestines.^1^ Although UC and CD have a chronic course, their etiology is unknown, and they are similar to each other due to their inflammatory nature, there are some differences depending on the regions involved in the gastrointestinal tract and the symptoms that occur. Crohn’s disease is characterized by transmural involvement anywhere in the gastrointestinal tract and causes fibrosis, obstruction, and irregular thickening of the bowel wall, whereas UC is characterized by limited involvement in the colon with continuous mucosal and submucosal inflammation.^1-3^ For this reason, experimental colitis models that are used in an attempt to understand the histopathological and morphological changes in the intestinal tract, to develop new therapeutic drugs for the treatment of disease, and to study the mechanisms of action of these drugs should be well defined.

Experimental colitis models published in the literature include the inducible colitis model, adoptive transfer model, spontaneous colitis model, transgenic colitis model, and genetically engineered animal models. Among these, the subgroup of chemically induced colitis model, one of the widely used models, includes colitis models induced by trinitrobenzene sulphonic acid (TNBS), dextran sodium sulfate, oxazolone, acetic acid, dinitrochlorobenzene sulphonic acid, indomethacin, iodoacetamide, carrageenan, and peptidoglycan-polysaccharide.^2^

An ideal colitis model should reflect the histological character and autoimmune nature of the disease and should show the same clinical findings as the human IBD. It needs to be simple, cost-effective, easily applicable, and reproducible. However, there is no ideal colitis model that completely explains the etiopathogenesis of IBD. Since the inflammation in the intestines does not occur in the same ratio as the course of the disease, it causes the misinterpretation or repetition of the experiment.^4^

In this study, we aimed to compare the acetic acid-induced model, TNBS-induced model, and the combined model in which acetic acid and TNBS both are used together biochemically and histopathologically.

## Materials and Methods

### Animals

The study was approved by the Animal Experiment Ethics Committee of the Abant İzzet Baysal University with the number 2015/02. Fifty-six male Wistar albino rats with a mean age of 2-4 months and a mean weight of 200-250 g were used in this study. Rat chows and water ad libitum were used for feeding the rats. The rats were randomly divided into 4 groups.

### Induction of Colitis

On the day of induction, under mild anesthesia with intramuscular ketamine (50 mg/kg, Ketasol, Richter Pharma AG, Wels, Austria), a 6F feeding tube was placed 8 cm proximal from the anus in the Trendelenburg position. Group 1 (control) received 1.5 mL of 0.9% physiological saline (PS), group 2 (acetic acid) received 1.5 mL of 4% acetic acid (Merck, Darmstadt, Germany), group 3 (TNBS) received 0.25 mL of 30 mg TNBS (Sigma, St. Louis, MO, USA) in 50% ethanol solution, and group 4 (combined) received 1.5 mL of 4% acetic acid and 10 minutes later 30 mg TNBS. All the groups were sacrificed on days 3 and 7 following colitis induction and were divided into 2 subgroups (*n* = 7). Initial and final weights of all the subjects were recorded.

### Sample Collection

Following colitis induction on days 3 and 7, all rats underwent midline laparotomy with 50 mg/kg ketamine anesthesia. Five milliliters of intracardiac blood sample was collected to get plasma. Total colectomy was performed and the location of 8-10 cm from the anus was washed with 0.9% saline and stored for biochemical analysis. The remaining colon segment was stored in 10% buffered ­formalin solution for pathological examination.

### Tissue Homogenization

The colon tissues (approximately 100 mg) were homogenized in ice-cold phosphate-buffered saline (pH 7.4); at 1 : 9 weight/volume (w/v) at 16 000 rpm for 5 minutes. The homogenates were used.

### Measurement of Biochemical Parameters

Plasma samples were assayed for serum amyloid (SAA) (MyBioSource, San Diego, Calif, USA) and myeloperoxidase (MPO) (Hycult Biotech, Uden, the Netherlands), and tissue homogenates for tumor necrosis factor-alpha (TNF-α) (eBioscience, San Diego, Calif, USA), interleukin 33 (IL-33) (R&D, Minneapolis, Minn, USA), and ST2 (MyBioSource) with enzyme-linked immunosorbent assay method. Tissue protein levels were measured with bicinchoninic acid assay and the results were put in proportion with tissue protein.

### Histopathological Examination

Tissue samples were fixated with 10% buffered formalin solution, embedded in paraffin, and stained with hematoxylin–eosin (H&E). The stained slides were evaluated in a blinded manner under light microscope based on the criteria listed in Table 1 which were modified from the criteria proposed by Koçak et al.^5^

### Statistical Analysis

Kolmogorov–Smirnov test was used to analyze whether the numerical changes were in correlation with normal distribution. Data with normal distribution were expressed as mean ± standard deviation whereas data without normal distribution were expressed as median ± standard error of mean. For parameters with normal distribution, the groups were evaluated with one-way analysis of variance. In parameters that were significant, the difference between the groups was analyzed with post hoc Tukey test. In parameters that did not have a normal distribution, following Kruskal–Wallis variance analysis, the difference between the groups was analyzed with Mann–Whitney *U test*. Mean weights of the groups at the beginning and at the end of the test were evaluated with dependent sample *t-*test. Exact *P* values were provided, the results were evaluated within 95% CI, and *P* <.05 was regarded as statistically significant.

## Results

When the means of weights at the beginning and at the end of the experiment were compared, weight loss was found to be statistically significant in TNBS and combined groups (*P*  < .05). In the acetic acid group, following the colitis induction, weight loss was found to be significant on day 3 (*P*  < .05), whereas weight gain was observed on day 7.

All the groups were evaluated among themselves on day 3 and day 7, and the results of all biochemical parameters were summarized in Table 2. The *P* values of the results of the comparisons of acetic acid, TNBS, and combined groups with the control group are shown in Table 3. When acetic acid, TNBS, and combined groups were compared among themselves, there was no difference biochemically or histopathologically between the groups that were sacrificed on day 3. In the same way, there was no difference between the TNBS group and the combined group sacrificed on day 7 for any parameters. For serum amyloid A (SAA), there were differences between the acetic acid group-TNBS group (*P*  = .026) and the acetic acid-combined group (*P*  = .001), and for ST2, there were differences between the acetic acid-TNBS group (*P*  = .022) and the acetic acid-combined group (*P*  = .01).

**Figure 1.**
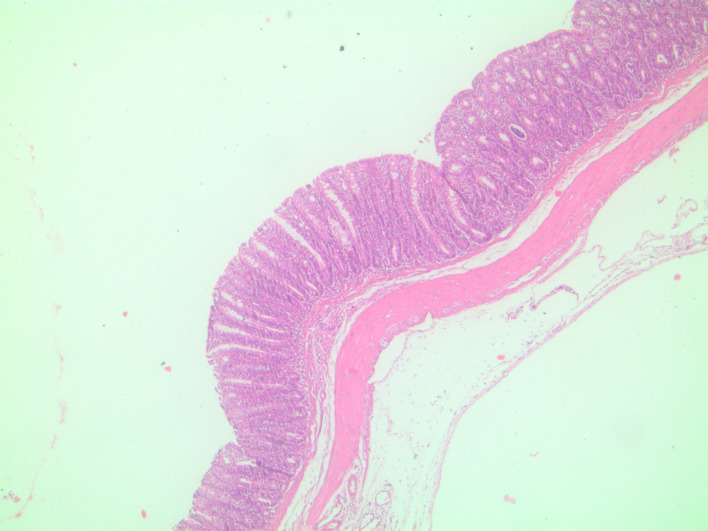
Acetic acid group on day 3, neutrophil-rich inflammatory cells in the lamina propria (H&E, ×40). H&E, hematoxylin and eosin.

**Figure 2.**
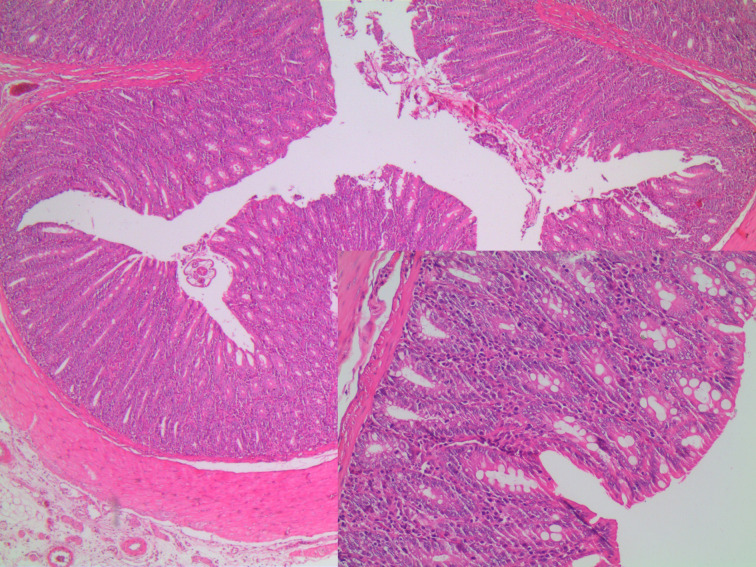
Acetic acid group on day 7, mild lymphocyte increase in lamina propria (H&E, ×40 and ×200). H&E, hematoxylin and eosin.

**Figure 3.**
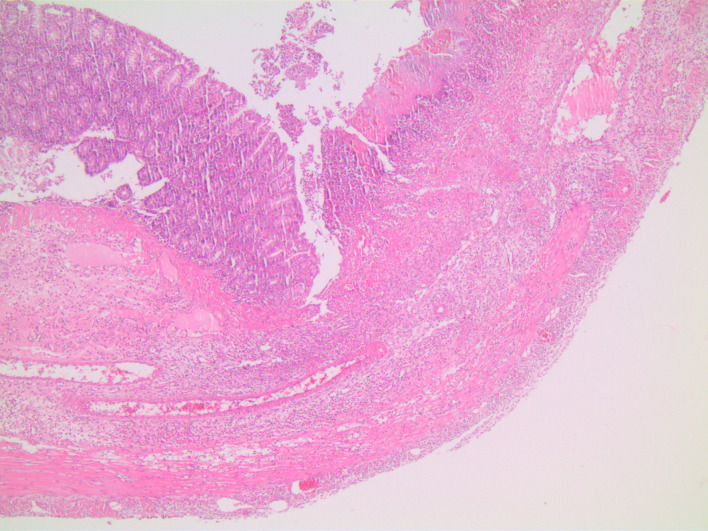
TNBS group on day 3, intact and necrotic mucosa areas, transmural inflammation, edema, and congestion (H&E, ×40). H&E, hematoxylin and eosin; TNBS, trinitrobenzene sulphonic acid.

**Figure 4.**
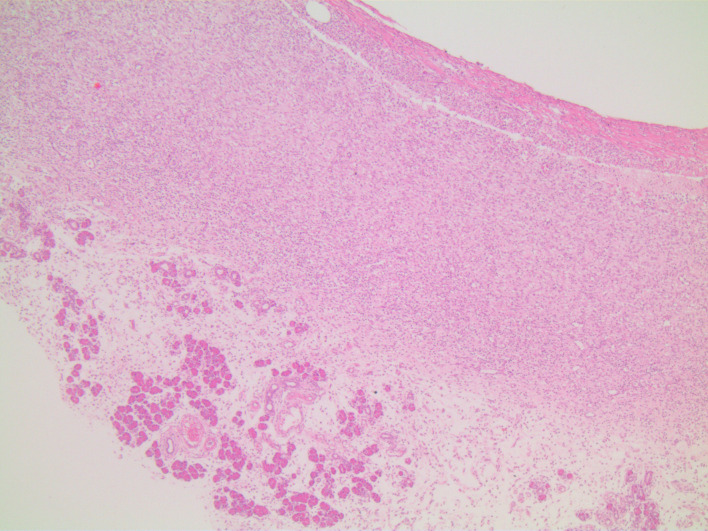
TNBS group on day 7, transmural inflammation, fibrosis, and adherence to pancreas (H&E, ×40). H&E, hematoxylin and eosin; TNBS, trinitrobenzene sulphonic acid.

**Figure 5.**
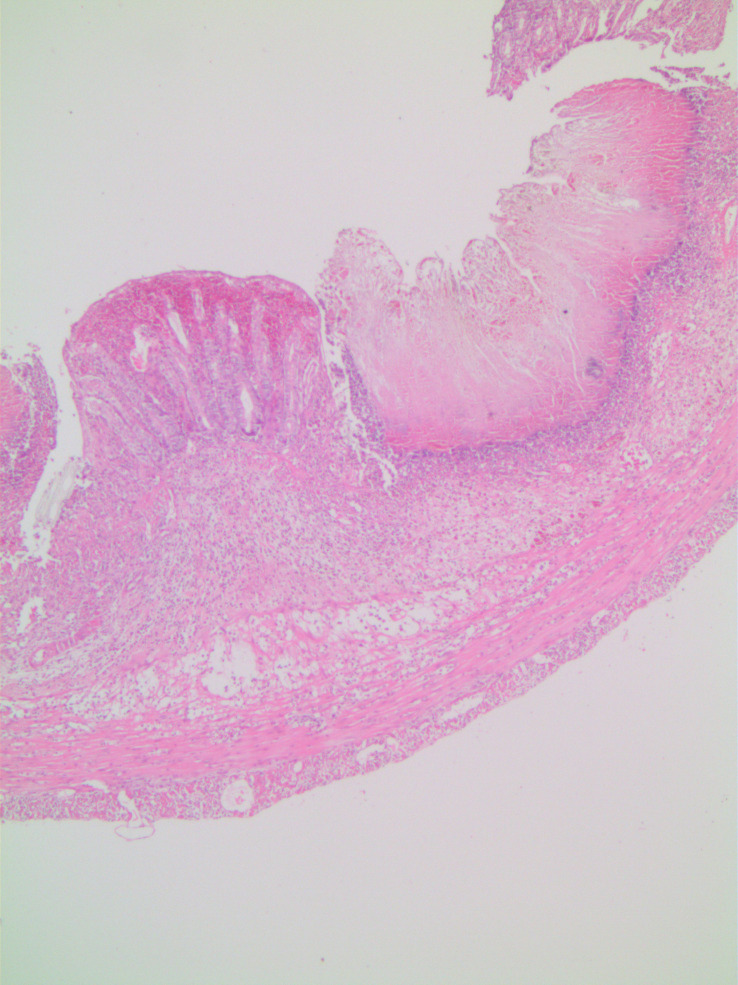
Combined group on day 3, edema, necrosis, and congestion (H&E, ×40). H&E, hematoxylin and eosin.

**Figure 6.**
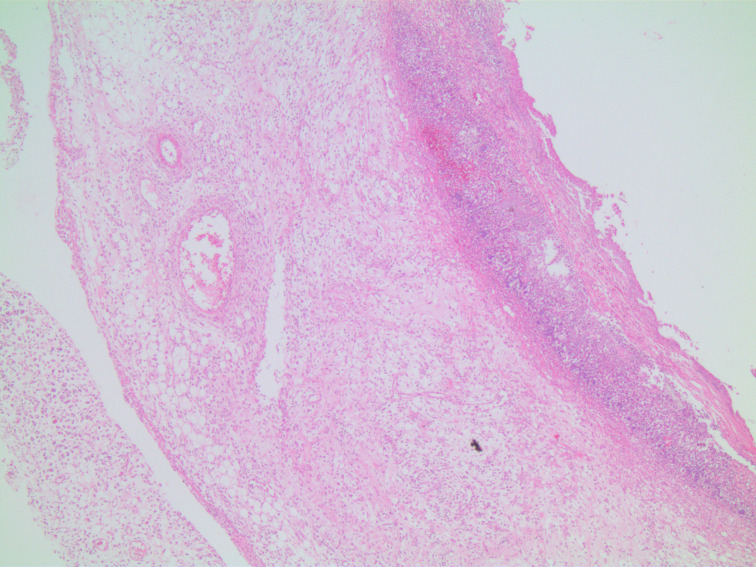
Combined group on day 7, extensive and deep ulceration and fibrosis (H&E, ×40). H&E, hematoxylin and eosin.

In microscopic damage scoring, when acetic acid group was compared with control group on day 7 (*P*  = .003) and when other experiment groups were compared with the control group, significant differences (*P*  = .001) were found. When the study groups were compared among themselves in pairs, there was no difference between groups sacrificed on day 3. There was no difference between TNBS group and combined group sacrificed on day 7, yet there were significant differences between acetic acid group and other groups (*P* values of .003 and .008, respectively). In the histopathological examination, on day 3, in the acetic acid group, there was mainly mucosal and submucosal inflammation observed (Figure 1), on day 7, the inflammation regressed and regenerative changes appeared (Figure 2). In TNBS and combined groups, on days 3 and 7, there was focal ulceration fields and transmural inflammation (Figures 3-6).

## Discussion

In this study, where experimental chemical colitis models were compared, acetic acid and TNBS were used together to come up with a new model; acetic acid, TNBS, and combined models were compared with the control group to study the success of the experimental groups by measuring the levels of plasma SAA, plasma MPO, colon TNF-α, colon IL-33, and colon ST2 levels together with histopathological scoring. In experimental colitis models, the study animals go through weight loss mainly due to inflammation and diarrhea. The acetic acid group lost weight on day 3, while it gained weight on day 7 due to clinical improvement of diarrhea and regression of inflammation. In the combined group, the study animals lost more than 10% of their body weight. These results indicated the presence of a significant inflammation in the colon in these groups.

In the colitis model induced by acetic acid, inflammation was seen in mucosa and submucosa. The inflammation is characterized by necrosis, vascular dilatation, and edema. The inflammation caused by acetic acid in the colon resembles UC histopathologically.^2^ Acetic acid causes colitis with acute effects. The inflammation starts to regress after 3-4 days and the chemical damage completely heals in days and in 2-3 weeks in rats.^6^ In this study in histopathological examination there were inflammation and necrosis fields in mucosa and submucosa in correlation with the literature and acetic acid-related inflammation spontaneously regressed by day 7 and improvement-related regenerative changes were seen. However, in certain drug efficacy studies, in the colitis model induced by acetic acid, experimental animals are sacrificed a week later to evaluate drug efficacy.^7-9^ In such studies conducted about drug efficacy on the acetic acid model, it is thought that evaluating the efficacy of the drug on days 2-3 where inflammation was significant would be more beneficial.

The TNBS-induced colitis model resembling CD features was first described by Morris et al.^10^ They showed transmural inflammation with granulomas and Langhan’s type giant cells as well as mucosa with “a cobble-stone” like appearance. In another study, TNBS was shown to have induced transmural inflammation resulting in diffuse necrosis, inflammatory granuloma, and neutrophil infiltration in the submucosa.^11^ Brenna et al^12^ showed the presence of cryptitis and crypt distortion in the biopsy samples of rats that had induced colitis with TNBS. In this study, the inflammation covered the whole layers like the case in CD; in other words, there was transmural involvement in TNBS groups. During the acute stage, there was mixed type inflammatory cell infiltration, edema, and congestion in mucosa and submucosa with the preponderance of neutrophils. During the chronic stage, the cellular infiltration had lymphocyte dominance together with fibrosis and fibrosis-related surrounding tissue adhesions.

There are previous studies by using acetic acid and dinitrochlorobenzene sulphonic acid together and by inducing “knock-out” models with chemicals.^6,13,14^ Ours is the first and only study in literature where colitis was induced by using acetic acid together with TNBS. When the combined group was compared with the control group histopathologically on days 3 and 7, the difference was of statistical significance. During the acute phase, the transmural inflammation observed in the mucosa and submucosa had a dominance of neutrophils whereas lymphocytes dominated during the chronic phase. Furthermore, there was an increase in intraepithelial lymphocytes, increase in the frequency of mitosis, bleeding, edema, widespread necrosis, and ulceration extending to the depths of the tissue. On day 7, there was fibrosis on the serosal surface and adhesions to the neighboring tissues. In the combined group, there were findings similar to those of CD in humans. While the combined group was shown to be histopathologically successful from the acetic acid group in the chronic period, no statistically significant difference was found with the TNBS group. However, further studies should be planned to observe how long the effects of the combined model last.

Serum amyloid A is accepted as an acute phase reactant which increases during the course of inflammatory conditions like IBD.^15^ Myeloperoxidase is a structural enzyme found in polymorphonuclear leukocytes and critical in innate immune responses, and it catalyzes the formation of hypochlorous acid which causes tissue damage at the site of inflammation.^16^ In this study, where experimental models are compared, SAA and MPO levels were used as systemic inflammation markers. When compared with the control group, in the acetic acid group, plasma SAA and MPO levels on days 3 and 7 did not show any significant difference. The acetic acid model mostly caused a local effect in the intestines. On day 3 of the TNBS group, SAA plasma levels were compared with the control group and were seen to have increased, yet this increase did not reach the level of statistical significance. On day 7 of the TNBS group, the increase in plasma SAA levels was found to be statistically significant. This can be explained by the TNBS model systemically showing a more chronic effect (Table 3).

When the role and effects of TNF-α in the inflammatory process are considered, it is seen as one of the most important proinflammatory cytokines in IBH. As it plays an instrumental role in triggering inflammation, an increase in TNF-α level is anticipated in induced colitis models.^17^ In studies conducted in recent years, IL-33/ST2 signal pathway was found and this was shown to be activated and increased especially during the acute phase of the inflammation in different autoimmune and inflammatory diseases like asthma, sepsis, trauma, and atherosclerosis. There are studies indicating activation of this pathway in IBD.^18-20^ In this study, the TNBS model and the combined model which was produced for the first time were shown to have increased levels of TNF-α, IL-33, and ST2 on days 3 and 7 in correlation with the literature. However, in the acetic acid group, despite there were increases in TNF-α, IL-33, and ST2 levels on day 3, statistically significant increases could not be shown on day 7 due to the regression of inflammation as was the case for other parameters.

The limitation of our study was that the long-term effects of the combined model formed with TNBS and acetic acid were not evaluated. A longer-term study will contribute to the examination of the effects of the combined model in the chronic period and the recovery process and to the development of the model.

In conclusion, acetic acid induced an inflammation with acute effect and the inflammation totally regressed within a week. The combined model was found to be more successful than models employing acetic acid or TNBS by itself as regards to inflammation. The combined model is a suitable experimental colitis model in which this pathway can also be evaluated.

## Figures and Tables

**Table 1.** Microscopic Damage Scoring

Evaluated Region		Score			
		**0**	**1**	**2**	**3**
Mucosal epithelium	**Intraepithelial lymphocyte**	None	Mild	Moderate	Severe
	**Ulceration**	None	Mild superficial	Moderate	Whole layer
Crypts	**Mitotic activity**	Lower 1/3	Mild mid 1/3	Middle 2/3	Upper 1/3
	**Mucus accumulation**	None	Mild	Moderate	Severe
Lamina propria	**Vascularity**	None	Mild	Moderate	Severe
	**Granulocyte accumulation**	None	Mild	Moderate	Severe
	**Mononuclear cell accumulation**	None	Mild	Moderate	Severe
Submucosa	**Edema**	None	Mild	Moderate	Severe
	**Granulocyte accumulation**	None	Mild	Moderate	Severe
	**Mononuclear cell accumulation**	None	Mild	Moderate	Severe
Fibrosis		Absent	Present		

**Table 2.** The Results of the Biochemical Parameters of Control, Acetic Acid, TNBS, and Combined Groups

		Control Group	Acetic Acid Group	TNBS Group	Combined Group	*P*
SAA (µg/mL)	**Third day**	0.86 ± 0.26	0.92 ± 0.36	1.14 ± 0.35	1.50 ± 1.44	.003
	**Seventh** **day**	0.71 ± 0.22	1.14 ± 0.16	1.65 ± 0.34	3.15 ± 0.63	.003
MPO (µg/mL)	**Third day**	6.36 ± 2.10	10.88 ± 1.25	13.34 ± 3.33	11.28 ± 3.09	.000
	**Seventh ** **day**	6.49 ± 1.88	9.36 ± 1.82	11.88 ± 4.09	13.52 ± 1.88	.000
TNF-α (pg/mg protein)	**Third day**	44.01 ± 5.11	122.4 ± 44.91	113.04 ± 19.65	165.08 ± 64.75	.02
	**Seventh day**	33.14 ± 5.89	58.26 ± 38.09	90.97 ± 8.21	107.03 ± 38.14	.02
IL-33 (pg/mg protein)	**Third day**	43.76 ± 11.54	775.91 ± 152.08	173.70 ± 63.31	488.70 ± 122.27	.010
	**Seventh** **day**	46.05 ± 16.14	273.03 ± 109.67	378.29 ± 95.42	323.15 ± 119.95	.010
ST2 (pg/mg protein)	**Third day**	5.4 ± 0.93	22.55 ± 1.84	21.63 ± 3.81	19.71 ± 3.12	.000
	**Seventh** **day**	3.47 ± 0.59	5.14 ± 0.75	11.60 ± 2.37	15.77 ± 6.73	.000

Results were presented as median ± standard error of mean.

SAA, serum amyloid A; MPO, myeloperoxidase; TNF-α, tumour necrosis factor alpha; IL-33, interleukin 33; ST2, IL-33 receptor; TNBS, trinitrobenzene sulphonic acid.

**Table 3.** Comparison of Study and Control Groups

		Control and Acetic Acid	Control and TNBS	Control and Combined
SAA	**Third day**	0.432	0.111	.048*
	**Seventh day**	0.268	0.030*	.003*
MPO	**Third day**	0.077	0.000*	.029*
	**Seventh day**	0.454	0.008	.000*
TNF-α	**Third day**	0.026*	0.001*	.017*
	**Seventh day**	0.073	0.008*	.005*
IL-33	**Third day**	0.026*	0.002*	.030*
	**Seventh day**	0.149	0.005*	.009*
ST2	**Third day**	0.001*	0.001*	.001*
	**Seventh day**	0.086	0.002*	.004*

*P  <.05 was regarded as statistically significant.

SAA, serum amyloid A; MPO, myeloperoxidase; TNF-α, tumour necrosis factor alpha; IL-33, interleukin 33; ST2, IL-33 receptor; TNBS, trinitrobenzene sulphonic acid.
