# Erratum

**DOI:** 10.5152/tjg.2022.231708

**Published:** 2023-09-01

In the Original Article by Cinpolat et al, entitled “A Chemically Induced Experimental Colitis Model with a Simple Combination of Acetic Acid and Trinitrobenzene Sulphonic Acid?” (Turk J Gastroenterol. 2023;34(3):196-202. DOI: 10.5152/tjg.2022.22174) which was published in the March 2023 issue of the Turkish Journal of Gastroenterology, it has come to our attention that an oversight occurred in accurately disclosing the funding information. The precise funding disclosure has been implemented on the online version of this article. You can access the corrected version of the article following the link provided below:

https://www.turkjgastroenterol.org/en/a-chemically-induced-experimental-colitis-model-with-a-simple-combinationof-acetic-acid-and-trinitrobenzene-sulphonic-acid-137028

